# Utilization of Birch Bark-Derived Suberinic Acid Residues as Additives in High-Density Fiberboard Production

**DOI:** 10.3390/ma18174171

**Published:** 2025-09-05

**Authors:** Julia Dasiewicz, Anita Wronka, Janis Rizikovs, Grzegorz Kowaluk

**Affiliations:** 1 Faculty of Wood Technology, Warsaw University of Life Sciences−SGGW, Nowoursynowska Street 159, 02-787 Warsaw, Poland; i002019@sggw.edu.pl; 2 Department of Technology and Entrepreneurship in Wood Industry, Institute of Wood Sciences and Furniture, Warsaw University of Life Sciences−SGGW, 02-787 Warsaw, Poland; anita_wronka@sggw.edu.pl; 3 Latvian State Institute of Wood Chemistry, LV-1006 Riga, Latvia; janis.rizikovs@kki.lv

**Keywords:** fiberboard modification, lignocellulosic materials, industrial bark residues

## Abstract

This study investigates the use of suberinic acid residues (SARs), derived from birch outer bark, as a bio-based additive in high-density fiberboard (HDF). Boards with target densities of 800 kg m^−3^ were produced with SAR contents of 0, 1, 5, 10, 20, and 50%. Standardized tests evaluated mechanical properties: screw withdrawal resistance, modulus of elasticity, modulus of rupture, and internal bond, as well as moisture resistance through surface water absorption, water absorption, and thickness swelling. Density profiles were also analyzed. SAR content influenced HDF performance in a concentration-dependent manner. The most notable improvements in mechanical properties occurred at 5% SAR, where fine particles likely enhanced internal bonding and stiffness. However, higher SAR levels led to reduced mechanical strength, possibly due to an excessive particle surface area exceeding the adhesive’s bonding capacity. Moisture resistance declined with increased SAR, attributed to its hydrophilic nature and process parameters, although SAR-modified boards still outperformed those with other biodegradable additives like starch. SAR also affected the density profile, improving core densification at moderate levels. Overall, SAR shows potential as a renewable additive for enhancing HDF performance, particularly at low concentrations, balancing mechanical strength and environmental benefits.

## 1. Introduction

Compared to medium-density fiberboard (MDF), high-density fiberboard (HDF), an engineered wood product, offers more density and strength since it is formed from highly compacted wood fibers [1]. HDF is used extensively in flooring, furniture, doors, and interior paneling because of its smooth surface, strength, and exceptional machinability [2]. In order to overcome its difficulties, changes are being investigated, such as adding mineral fillers and industrial byproducts, which can increase sustainability, lower formaldehyde emissions, and improve mechanical qualities.

Scientists are increasingly seeking natural ingredients to support innovative product development [3]. In recent years, tree bark—which is rich in bioactive compounds that can be easily extracted using various chemical methods—has regained popularity. Its antioxidant, antibacterial, and anti-inflammatory properties are attributed to a range of bioactive substances, including phenolics, flavonoids, tannins, and terpenoids [4,5,6]. For example, maritime pine bark extract—known as pycnogenol—is valued for its health benefits, while pine bark is also used in wood adhesives and as a sustainable source of fuel. Additionally, bark may be used to make carbon materials, glues, culinary additives, and medical supplies [7]. Among various types of tree bark, birch bark stands out due to its high content of valuable bioactive compounds, particularly betulin and betulinic acid, which are primarily concentrated in the outer bark [8]. These triterpenes exhibit diverse pharmacological activities such as anticancer, anti-inflammatory, antiviral, and liver-protective effects, which makes them promising compounds for use in the pharmaceutical, cosmetic, and food industries [9]. To optimize the extraction of these compounds, several methods have been explored, such as solvent-based techniques using ethanol or methanol, green solvents like choline chloride with lactic acid, and pressurized hot water extraction, each offering distinct advantages depending on the target compound [10]. Mechanical and hydrostatic separation methods are also employed to enhance raw material quality by efficiently isolating bark from bast fibers. Beyond triterpenes, birch bark contains additional active substances like lupeol and γ-sitosterol, further expanding its potential [11]. Extracts from birch bark are used in commercial wound-healing products, as well as in skincare formulations aimed at managing inflammation, acne, and signs of aging [12]. Beyond biomedical applications, components such as suberinic acids (SAs) derived from birch bark have also demonstrated significant potential in the field of wood protection. Recent studies have shown that various SA-based formulations, particularly ethanol-dissolved fractions, can effectively impregnate wood, enhancing its resistance to moisture and fungal degradation [13]. These versatile applications highlight birch bark’s growing relevance as a renewable and functional natural resource. Each of these extraction methods, however, generates a significant amount of post-extraction waste, which, according to circular economy principles, should be repurposed for further applications to maximize resource efficiency and minimize environmental impact.

Bark post-extraction waste, which comprises residues left after the extraction of bioactive compounds, can be used in various applications, such as energy utilization. It is suitable for the production of solid biofuels like pellets or briquettes [14]. Or it can be used in pyrolysis to produce biochar or bio-oil [15]. Another potential application of post-extraction waste is its use as sorbents for wastewater treatment due to its ability to adsorb heavy metals and other pollutants thanks to its porous structure [16,17]. However, it should be noted that birch outer bark used for suberinic acid extraction has properties that reduce formaldehyde emissions [18,19]. Similar properties are demonstrated by activated carbon [20,21] or green tea leaves [22]. Depending on the type of additive, it may exhibit different functions—some may contribute to formaldehyde reduction, and others may lower the flammability of wood composites [23] or reduce surface absorption, serving as a substitute for paraffin emulsion, such as beeswax [24].

Previous studies on the use of post-extraction waste, like suberinic acid residues (SARs), have focused on incorporating these residues into OSB and particleboards, where an attempt was made to use post-extraction waste as a binder without additional chemical additives. Boards with a nominal density of 700 kg m^−3^ were produced using three different particle fractions, which are typically used in the outer and inner layers of particleboards and in OSB boards. Unfortunately, the mechanical properties of the tested boards did not meet most standards, which is why subsequent studies explored alternative ways of incorporating post-extraction residues [25]. The addition of SAR as a filler in plywood provided a dual benefit, as it accelerated the gelation time and served as a substitute for commercially used rye flour. A higher density of glue lines was observed, which positively influenced the strength parameters, particularly in the case of hot-pressed plywood, and the results obtained were comparable with the reference variant [26]. Due to its powdery form, the application of SAR may be debatable; however, given the benefits it offers, it is certainly worth considering. Depending on the form of SAR implementation, it can negatively affect the structure of wood composites. The powdered form contributed to the deterioration of strength parameters. The SAR content was 0, 5, 10, and 15%, with dimensions of 0.250 mesh [27]. At the same time, the use of different fractions as an additive to particleboard helped to offset these differences in terms of strength parameters. In this research, fractions between the following sieves were used: 1–0.25, 2–1, 5–2, and 8–5 mm [25]. The literature provides examples of fine-particle chemical materials successfully incorporated into fiberboard structures. One such example is wollastonite—a mineral composed of calcium and silica, which can act as a resin extender in MDF. Panels with a 9:1 ratio of urea-formaldehyde (UF) to wollastonite exhibited properties comparable to those containing only UF, with the exception of internal bond strength [28]. Other mineral fillers, such as calcium carbonate, talc, and kaolin, are also widely used to enhance the processing efficiency, cost-effectiveness, and overall quality of fiber-based materials. These additives contribute to improved production rates and surface properties, including optical appearance. However, their incorporation may lead to a slight reduction in mechanical strength, which must be carefully considered when optimizing fiberboard formulations [29]. Eco-friendly additives are increasingly used to enhance fiberboard properties while reducing environmental impact. Calcium lignosulfonate, combined with 3.5% PF resin, enables a reduction in resin content without compromising performance, with optimal results at ≤10% addition [30]. Similarly, ammonium lignosulfonate, used with UF resin, lowers formaldehyde emissions while maintaining suitable physical and mechanical properties, making it a viable alternative for sustainable HDF production [31]. To further enhance the composition of fiberboard, additional raw materials, in addition to bio-based adhesives, are being investigated. Incorporating activated carbon into MDF formulations has been demonstrated to alter its physical characteristics, perhaps providing advantages in specific applications [32]. Zeolite, a mineral that increases bending strength and the elastic modulus and improves mechanical performance, is another interesting additive. However, its application has drawbacks, as it may marginally weaken internal bonds while increasing thickness, swelling, and water absorption [33]. A review of the literature reveals a notable research gap, as the use of SAR in fiberboards remains uncommon, despite its potential to positively influence the properties of HDF due to the unique characteristics of the material.

The purpose of this research was to test the effect of post-extraction birch bark waste–SAR on the structure of HDF and its physical and mechanical properties. In the scope of the research, the wooden fibers have been substituted with SAR waste in the amount of 5–50% (*w*/*w*) during HDF production. To evaluate the effect of such a substitution, the modulus of rupture, modulus of elasticity, internal bond, and screw withdrawal resistance have been tested, as well as the density profile, thickness swelling, surface water absorption, and water absorption.

## 2. Materials and Methods

### 2.1. Materials

**Figure 1:**
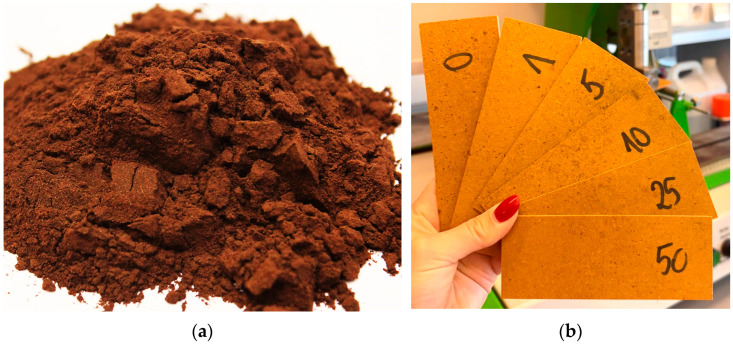
The SAR powder used in panel production (**a**) and produced panels before the bending test (**b**) (the numbers on the samples are the proportions (*w*/*w*) of suberinic acid residue).

High-density fiberboard (HDF) was produced under controlled laboratory conditions using industrial fibers derived from pine (*Pinus sylvestris* L.) and spruce (*Picea abies* (L.) H.Karst), supplied by IKEA Industry Poland Sp. z o.o., Orla, Poland. The roundwood used for fiber production typically consists of 2.4 m long logs with a 7–12 cm diameter at the smaller end. The fibers were dried to an approximate moisture content of 4%. The HDF panels were bonded with an UF resin (Silekol S123, Silekol Sp. z o.o., Kędzierzyn-Koźle, Poland), characterized by a formaldehyde-to-urea (F:U) molar ratio of 0.89, a pH value of 9.6, a viscosity of 470 mPa·s, and a resin content of 12%, which aligns with common industrial practices. The SAR after alkaline-ethanol depolymerization of extracted birch outer bark was delivered by the Latvian State Institute of Wood Chemistry (Riga, Latvia) and has been described in detail by Makars et al. [34]. In general, to obtain SAR, birch outer bark undergoes two sequential ethanol extractions, followed by air-drying at ambient conditions. The dried material was depolymerized in a KOH solution for 60 min at 66–80 °C, depending on the solvent system. After cooling, the reaction mixture was filtered, and the solid fraction was dispersed in water (100 g L^−1^), acidified to pH 2.0 with HNO_3_, and subsequently filtered and rinsed with deionized water. The fraction smaller than 0.25 mm of SAR has been used in the production of panels (Figure 1a). That fraction has been sieved from SAR dried and milled in a laboratory Retsch SM 100 knife mill (Retsch GmbH, Haan, Germany) before sieving.

### 2.2. Preparation of Panels

The study used laboratory-produced high-density fiberboards (HDFs), formed in a dry process. Each board had a target density of 830 kg m^−3^, dimensions of 320 × 320 mm, and a nominal thickness of 3 mm (Figure 1b). Two replicate samples were prepared for each board type. The experimental series included a control variant (without SAR) and boards containing different proportions of suberinic acid residue (SAR) at 5%, 10%, 25%, and 50% by total panel weight, referred to as SAR5, SAR10, SAR25, and SAR50, respectively. SAR was incorporated during the resination step. Prior to adhesive application, the wood fibers were divided into three layers: an inner layer (68% by weight) and two outer layers (each 16%). SAR was applied only to the inner layer fibers. The mats were manually formed and hot-pressed using a hydraulic press (AKE, Mariannelund, Sweden) at 180 °C, with a pressing duration of 20 s per millimeter of thickness (60 s total) and a maximum pressure of 2.5 MPa [35]. After pressing, the boards were conditioned at 20 °C and 65% relative humidity until a constant weight was achieved.

### 2.3. Characterization of the HDF Panels

**Figure 2:**
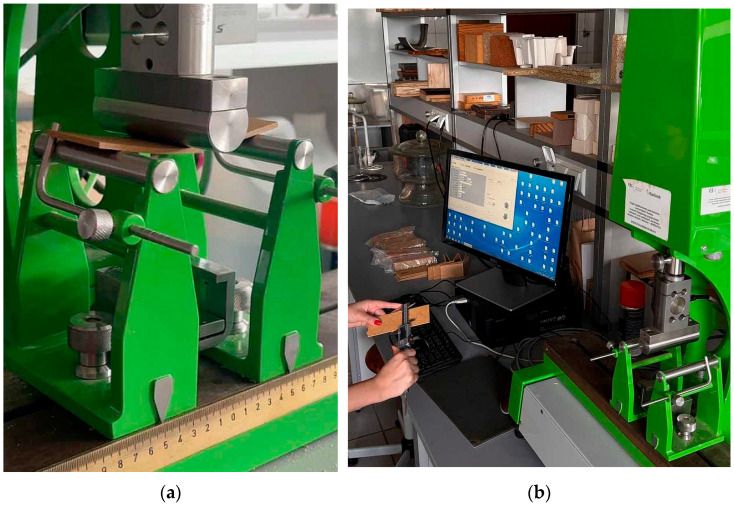
Measurement of MOR and MOE on a computer-controlled testing machine (loaded sample during bending (**a**) and entire stand view during measuring of the sample dimensions (**b**)).

Panel properties were assessed according to relevant European Standards and included measurements of density [36], the modulus of rupture (MOR) and modulus of elasticity (MOE) (Figure 2a) [37], internal bond strength (IB) [38], and screw withdrawal resistance (SWR) [39]. Additionally, water absorption (WA), thickness swelling (TS) after 2 and 24 h of immersion [40], surface water absorption (SWA) [41] were evaluated. For each panel type, at least 12 samples were tested, except for SWA, where two samples per type were examined. To assess density profiles, three specimens of 50 mm × 50 mm were extracted from each panel type. Based on preliminary results, one profile per type was selected for detailed analysis. Density distribution through the panel thickness was determined using a Grecon DA-X X-ray densitometer (Grecon, Alfeld, Germany) with a scanning resolution of 0.02 mm. All mechanical tests were performed on a computer-controlled universal testing machine (Figure 2b) (delivered by Research and Development Centre for Wood-Based Panels, Czarna Woda, Poland). Where applicable, obtained values were compared with the corresponding requirements specified in European Standards [42].

### 2.4. Statistical Analysis

Statistical analysis of the mean values for various factors and levels was carried out using analysis of variance (ANOVA) and Student’s t-test at a significance level of α = 0.05. When appropriate, Duncan’s multiple range test was used for further comparison. These analyses used IBM SPSS Statistics (IBM SPSS 20, Armonk, NY, USA). The letters “a”, “b”, “c”, etc., in the plots indicate statistically homogenous groups.

## 3. Results and Discussion

### 3.1. Modulus of Rupture and Modulus of Elasticity

**Figure 3:**
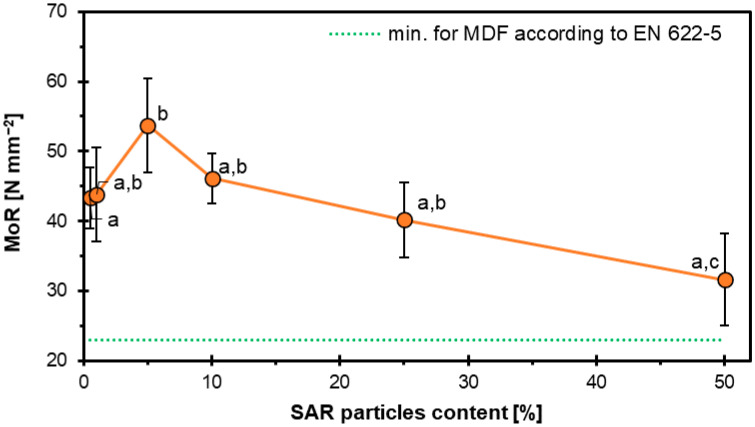
Influence of SAR content on the MOR of HDF boards.

The results of the influence of SAR content on the modulus of elasticity of the HDF boards are shown in Figure 3. The highest value was shown by sample SAR5 (53.7 N mm^−2^), and then the samples began to decrease as follows: SAR10-46.2 N mm^−2^, SAR25-40.2 N mm^−2^, SAR50-31.6 N mm^−2^. Samples SAR0 (43.3 N mm^−2^) and SAR1 (43.9 N mm^−2^) showed similar properties, which proves the insignificant effect of the 1% SAR additive on MOR properties.

**Figure 4:**
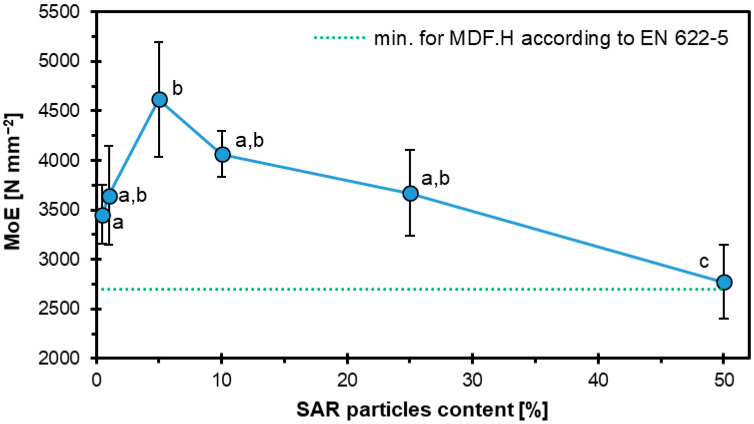
MOE variation in HDF boards with different SAR content levels.

Figure 4 shows the variation in the MOE in HDF boards with different amounts of SAR content. As in the MOR test, all samples were referred to EN 622-5 [42]. The highest value also came out for SAR5 of 4621 N mm^−2^, and then, as in the MOE study, the samples successively decreased: SAR10-4066 N mm^−2^, SAR25-3671 N mm^−2,^ and SAR50-2771 N mm^−2^. The SAR50 sample, which was the sample with the highest SAR content, showed the lowest properties and was on the borderline of the norm, which was 2700 N mm^−2^. Similar characteristics to sample SAR25 were displayed by sample SAR1 (3646 N mm^−2^), whereas sample SAR0 had a value of 3455 N mm^−2^.

Using another powdered HDF additive in the form of starch resulted in increased MOR and MOE properties. The potato starch was added in proportions of 0, 1, 5, 10, and 20%. The highest MOE value (3900 N mm^−2^) was observed with 20% starch. Similarly, samples with 20% starch addition showed the highest MOR value, which was about 138% higher compared to the reference sample [43]. The values of MOR and MOE also increased in the case of rice starch [44].

In the case of plywood, MOE and MOR results increased with an increase in SAR filler additive [26]; as the particleboard increased the SAR addition content, MOE and MOR values decreased [27]. So, the addition of SAR, depending on its proportion and the type of wood-based composite, can affect the variation in MOR and MOE properties. In the case of HDF, despite a decrease in MOR and MOE values with an increase in the proportion of SAR, the study showed very high values, higher than those of the reference sample. In contrast to other organic powders [43,44,45], the use of powdered SAR in HDF boards shows significant potential.

### 3.2. Screw Withdrawal Resistance

**Figure 5:**
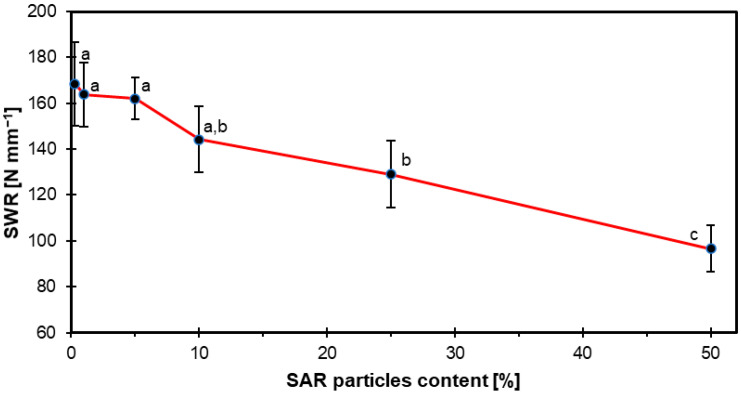
SWR of HDF boards as influenced by varying SAR content.

Figure 5 shows the SWR test results. As we can see from the graph, as the SAR content increases, the sample values decrease as follows: SAR0-169 N mm^−2^, SAR1-164 N mm^−2^, SAR5-162 N mm^−2^, SAR10-144 N mm^−2^, SAR25-129 N mm^−2,^ and SAR50-97 N mm^−2^. For the 5% and 10% SAR additions, the decrease in values was not significant compared to the reference sample.

In a study of particleboard with SAR addition, Maksymiuk et al. [27] observed that SWR values decrease as the content of this addition increases, with a significant decrease only from 5% of its proportion. Interestingly, samples with 5% (127 N mm^−1^) and 10% (113 N mm^−1^) SAR additive had higher SWR values compared to a reference sample with 0% of it (104 N mm^−1^). Nevertheless, the use of 15% (90 N mm^−1^) SAR additive resulted in lower SWR values.

This decrease in SWR value could be due to the addition of small particles, as demonstrated by Fehrmann et al. [46] while testing particleboard.

### 3.3. Internal Bond

**Figure 6:**
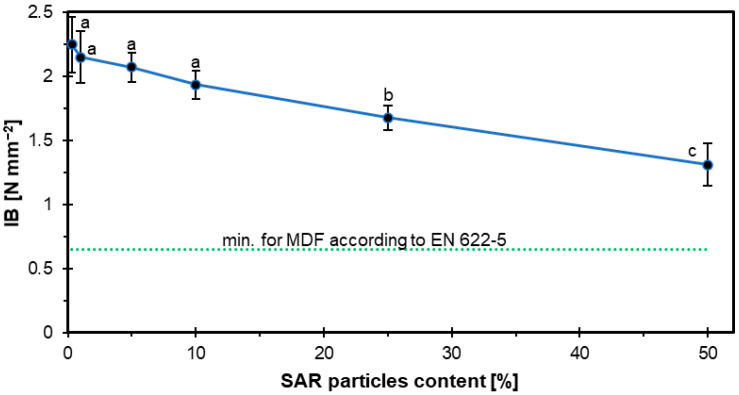
The IB of HDF boards as a function of SAR content.

The results for the IB of HDF boards with several proportions of SAR are shown in Figure 6. With the addition of SAR, the mechanical properties decrease, but still, those of all samples are high enough that they comply with EN 622-5 [42]. The samples decreased as follows: SAR0-2.25 N mm^−2^, SAR1-2.15 N mm^−2^, SAR5-2.07 N mm^−2^, SAR10-1.93 N mm^−2^, SAR25-1.68 N mm^−2^, SAR50-1.31 N mm^−2^.

Research by Wronka and Kowaluk [25] proved that reinforcing particleboards with SAR resulted in an improvement in their IB, especially the smaller fractions. The highest results came out for the 5/2 fraction, achieving up to 2.10 N mm^−2^. These observations suggested that SAR can positively influence the bond strength inside the boards, potentially confirming that the results can also show good properties for IB in HDF boards. Meanwhile, a study by Maksymiuk et al. [27] showed that adding SAR to particleboard showed different effects on IB. For particleboard with an up to 10% SAR addition, they observed an increase in perpendicular-to-surface (IB) tensile strength. However, moving beyond this to 15% SAR content resulted in a statistically significant reduction in IB values, below the required European standards. This means that only a well-chosen fraction and proportion can result in improved IB values.

The addition of lignin to the HDF boards in the form of a natural glue made from wheat straw, which was an agricultural residue, showed improved strength values for IB. Boards with 15% lignin content achieved the highest IB of 1.46 MPa [47]. The same increase in IB values came out when boric acid and borax decahydrate were added to urea-formaldehyde resin in MDF boards [48].

So, compared to other tests, in our tests also, despite the decrease in values with the increase in SAR addition, the results of the studied plates showed high enough IB values. They are much higher than the standard requirements, and compared to other tests using organic residues.

**Figure 7:**
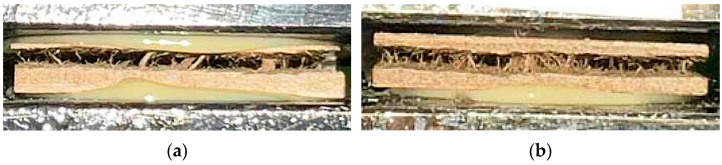
Various examples of a sample break during IB test: in the face zone (**a**) and in the core (**b**).

The observed deterioration of mechanical properties at SAR contents above 5% can be explained by two factors. First, fine SAR particles increase the overall surface area within the fiber mat. Since the adhesive amount was constant, its distribution becomes insufficient to ensure effective fiber-to-fiber bonding, leading to reduced cohesion. Second, at higher SAR concentrations, particles may form agglomerates, disrupting the homogeneity of the fiber network and creating local stress points. Both effects contribute to the reduction in bending strength (MOR, MOE) and the internal bond observed for SAR contents of 10% and higher. Similar behavior has been reported in studies where fine-particle additives exceeded their optimal concentration, limiting the efficiency of adhesive bonding in wood-based composites [27,46]. The examples of breaking the samples after the IB test are displayed in Figure 7.

### 3.4. Surface Water Absorption

**Figure 8:**
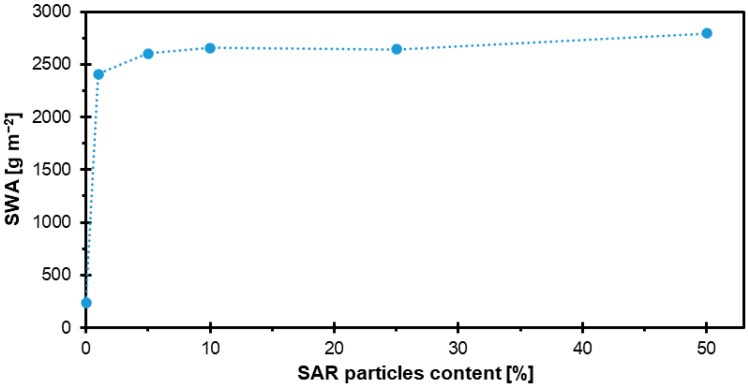
SWA of HDF boards with varying SAR contents.

The results of the SWA tests of HDF boards with different SAR contents are shown in Figure 8. The lowest surface absorption came out for SAR0 (238 g m^−2^) without the addition of SAR. But even a 1% addition of SAR increased absorption because it reached 2406 g m^−2^ for sample SAR1, and with further increases in SAR content, absorption increased as follows: SAR5-2604 g m^−2^, SAR10-2656 g m^−2^, SAR25-2641 g m^−2^, and SAR50-2794 g m^−2^.

Another natural ingredient used in the production of HDF was rice starch as a binder. The addition of starch to the boards was in amounts of 0%, 10%, 15%, and 20%. It was observed that as the proportion of starch increased, the SWA value decreased. The highest surface water absorption of 8611 g m^−2^ was recorded for boards with 10% starch addition, while the lowest, 4155 g m^−2^, was recorded for boards containing 20% starch [44]. Similar studies, using soybean starch as a binder in the production process of HDF boards, provided similar results. In this case, the following starch contents were used: 0%, 10%, 12%, 15%, and 20%. In general, the results showed a tendency for SWA values to decrease as the addition of soy starch increased, just as in the case of rice starch. The exception was the sample with 12% starch addition, which achieved a significantly higher water absorption value of 6263 g m^−2^. The lowest SWA value (3349 g m^−2^) was recorded for samples with 10% starch addition, while the highest proportion of starch (20%) resulted in the lowest SWA value of 2536 g m^−2^ [49].

In summary, the use of SAR in HDF resulted in relatively low surface water absorption values. The literature data for starch-modified HDF [43,44] indicate higher absorption values than those observed in this study; however, these results were obtained under different experimental conditions. Therefore, while SAR shows potential as an additive to limit water uptake, direct comparative studies are required to fully confirm its advantage over starch.

### 3.5. Water Absorption and Thickness Swelling

**Figure 9:**
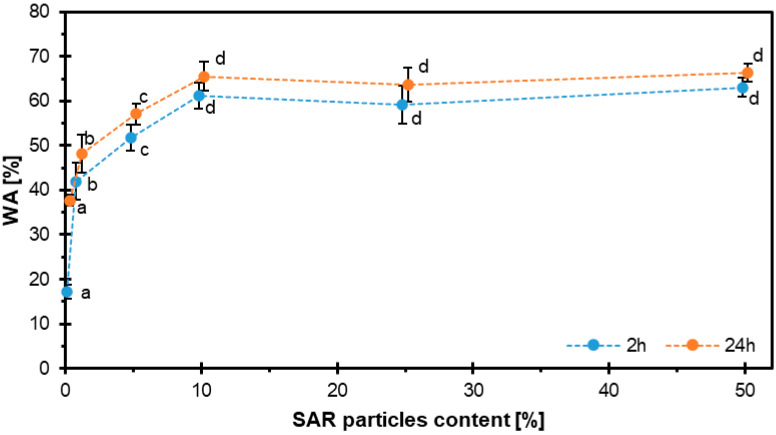
WA of HDF boards containing different SAR contents, measured after 2 h and 24 h of water immersion.

Figure 9 shows the WA of HDF boards with different SAR contents, measured after 2 h and 24 h of immersion in water. In both cases, temporal absorption increases with the addition of SAR content up to 10%, and further increasing it does not significantly affect the subsequent growth in WA. The results of the samples after a two-hour soaking time are as follows: SAR0-17.22%, SAR1-41.94%, SAR5-51.71%, SAR10-61.22%, SAR25-59.23%, and SAR50-63.11%. The increase in WA after 24 h is relatively uniform compared to the results obtained after 2 h.

**Figure 10:**
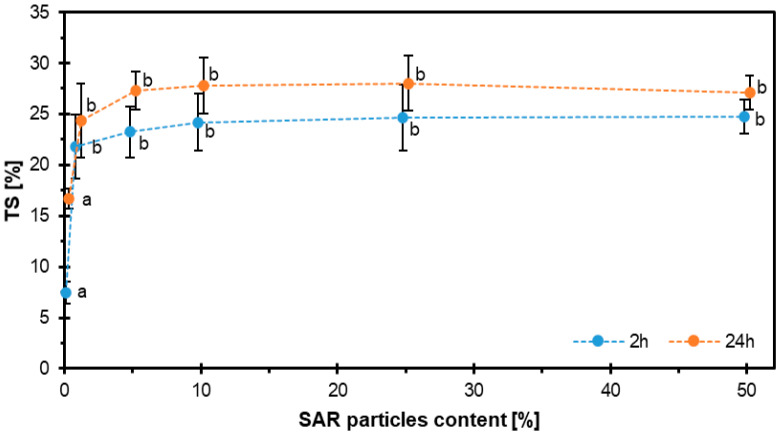
TS behavior of HDF boards with varying SAR contents after 2 h and 24 h water immersion.

The results of the TS tests are shown in Figure 10. As in WA, thickness swelling increases with the addition of SAR up to 10%, and no significant changes were observed thereafter. For samples soaked for 2 h, the results came out as follows: SAR0-7.45%, SAR1-21.81%, SAR5-23.26%, SAR10-24.19%, SAR25-24.64%, and SAR50-24.73%. For samples soaked for 24 h, the results were similar.

Smaller SAR particles of 1/0.25 and 2/1 fractions were observed to reduce WA after a 2 h of immersion for particleboard studies. Conversely, larger SAR fractions led to an increase in WA after 24 h of water exposure [25]. And in the study of Maksymiuk et al. [27], the use of SAR in particleboard caused an increase in WA with an increase in SAR; however, 5% (96.34%) and 10% (108.27%) of this additive is less than the reference sample (112.90%).

Other studies’ findings showed that chemical changes, such as acetylation, can significantly reduce the TS and WA of wood-based composites. For instance, fibers treated with acetylation exhibited the lowest levels of WA and TS in polypropylene composites containing wood powder compared to unmodified materials [50]. Similarly, pretreatment of wood chips with oxalic acid before refining has been shown to improve dimensional stability and reduce TS content in MDF [51].

MDF boards’ WA and TS were decreased by adding activated carbon, which was made from the dust produced by MDF shearing, to UF resin [52].

Higher-density boards tend to have reduced WA [53]. This trend suggests that increasing the density of HDF boards with SAR could potentially reduce WA.

### 3.6. Density Profile

**Figure 11:**
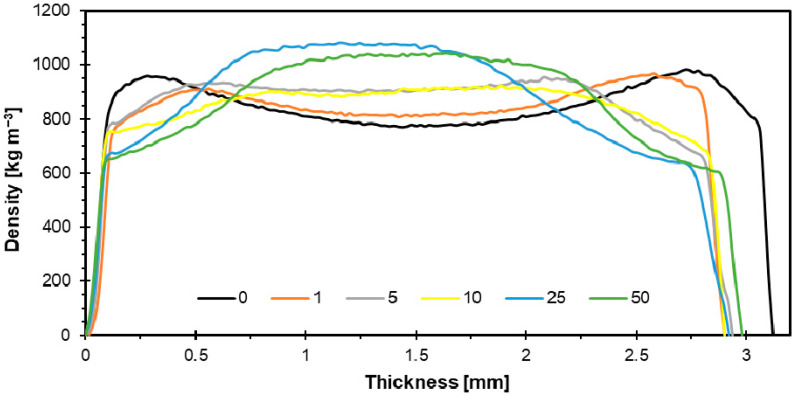
Representative density profiles of HDF boards containing different contents of SAR.

Figure 11 shows the density profile of the tested HDF samples containing different contents of SAR. As the addition of SAR in the board increased, the density profile in the middle part became larger and visually became more convex. For sample SAR1 (807 kg m^−3^), the density profile was most similar to reference sample SAR0 (793 kg m^−3^). Samples SAR5 (903 kg m^−3^) and SAR10 (909 kg m^−3^) gave a flat density profile, while SAR25 (1082 kg m^−3^) and SAR50 (1040 kg m^−3^) gave the largest and most convex density profiles.

Heat transmission, moisture movement, and internal pressure development are some of the elements that impact the formation of the vertical density profile in HDF boards during the hot pressing process. The density of the surface layers is often higher than that of the core [54,55,56]. The mechanical performance of HDF boards is significantly influenced by their density distribution features. Better internal integration and higher bending resistance (both MOR and MOE) are often associated with higher outer layer densities. However, the density of the board’s midsection also has a significant impact on the board’s overall functioning and IB [53]. The surface quality of HDF boards may suffer from variable density distribution, particularly if further processing is applied. The final board finish may be impacted by the altered surface roughness caused by the outer layers’ higher density [51,54]. HDF boards typically have densities between 810 and 1117 kg m^−3^. Boards with higher densities are often found to have better mechanical qualities, such as MOE and MOR [55]. It has been noted that adding SAR to particleboard changes the density distribution inside the material. SAR particles that are especially fine have a tendency to increase the outer layers’ density, which might improve the surface strength but could have a detrimental effect on the core’s homogeneity. SAR can be employed without substantially altering the density features of boards that include it, since the density values of these boards stay within acceptable bounds [22,24]. Organic fillers, such as starch, can change the density profile; mechanical properties are usually improved by increasing the binder content [35]. Although the type and amount of additives used can affect the final properties, HDF with organic additives usually has a consistent density profile [56,57,58,59].

## 4. Conclusions

The research showed that SAR addition significantly influences the physical and mechanical properties of HDF boards, with effects strongly dependent on concentration. The best results were achieved at 5% SAR, with peak MOE (4621 N mm^−2^) and MOR (53.7 N mm^−2^), indicating that small amounts of fine SAR particles can enhance internal structure and strength. Above this level, properties declined due to an increased particle surface area exceeding the adhesive’s bonding capacity. Similar trends were observed for SWR and IB, with up to 5–10% SAR maintaining acceptable performance, confirming the material’s suitability for practical use. Increased SAR content negatively affected water resistance, with SWA, TS, and WA rising significantly even at low concentrations and stabilizing above 10%. This was likely due to SAR’s partial hydrophilicity, which increased moisture absorption sites. Higher TS values at lower pressing temperatures (e.g., 180 °C) may result from insufficient thermal activation rather than water affinity. Pressing at around 248 °C improves SAR decomposition and bonding, enhancing water resistance and reducing TS [34]. Despite their increased water uptake, SAR-modified boards outperformed those with other organic additives like starch. SAR also improved core densification, potentially enhancing internal cohesion, although higher contents may reduce surface uniformity.

In summary, the use of SAR at levels up to 5% represents an effective method of enhancing the mechanical properties of HDF boards without significantly compromising other parameters. Beyond this value, a clear trade-off emerges between mechanical strength and moisture resistance, which should be taken into account when designing materials for specific applications. The findings confirm that SAR can serve as a functional bio-organic additive in the production of wood-based materials, combining good mechanical performance with relatively favorable hygroscopic properties. While SAR-modified boards demonstrated relatively low surface water absorption compared to the literature values reported for starch-modified boards, this comparison is indirect. Future studies should include direct comparative experiments under identical processing conditions to fully validate this observation.

While previous studies have indicated the potential of SAR to reduce formaldehyde emissions due to its natural composition and acidic functional groups, this work did not investigate formaldehyde release. Future research will focus on the quantitative analysis of emissions to fully assess SAR’s benefits in producing environmentally friendly HDF panels.

## Data Availability

The original data presented in the study are openly available in RepOD at https://doi.org/10.18150/SX3JX8 (created and accessed on 7 July 2025).
